# Roller Coaster Ride of Teaching Faculty under Pakistan Medical Commission

**DOI:** 10.12669/pjms.38.4.6021

**Published:** 2022

**Authors:** Afifa Ehsan, Ali Raza

**Affiliations:** 1 Dr. Afifa Ehsan, BDS, M.Phil, CMT, CME, MHPE (Scholar). Department of Oral Biology, Faryal Dental College, Lahore, Pakistan; 2 Dr. Ali Raza, BDS, M.Phil, PGD (Bioethics). University of Health Sciences, Lahore, Pakistan

The regulatory framework for Pakistan’s healthcare is evolving. However, ample gaps arise between anticipated objectives and real-world outcomes, thus reducing the utility of such decisions and necessitating various interim adjustments as stop-gap arrangements. In September 2020, Pakistan Medical Commission Act, 2020 was enacted by parliament, which finally replaced erstwhile Pakistan Medical and Dental Council (PM&DC).^1^ A tug of war spread over years thus came to an end. However, the origin of the Pakistan Medical Commission (PMC) can be traced back to an akin ordinance titled “Pakistan Medical Commission Act, 2019”, promulgated through a Presidential Order in October 2019. The regulatory policy and approach of decision-makers towards healthcare had started taking shape since then. Although PMC witnessed a short-lived standstill owing to legal and constitutional affairs during mid-2020, many other fault lines still exist and its overall performance is suboptimal by international standards.

The PMC Act was designed for regulation and control of the medical profession and to establish a uniform minimum standard of basic and higher medical education and training and recognition of qualifications in medicine and dentistry. The Commission comprises of three bodies namely the Medical and Dental Council (MDC), National Medical and Dental Academic Board (NMDAB), and National Medical Authority (NMA). The solitary provision related to teaching faculty in the Act provides that the standard and structure of faculty shall be regulated either by the Higher Education Commission or the affiliating university subject to the minimum standards determined by the Board. Diluting specific functions of regulation of faculty among three disjointed bodies will only hamper the safeguarding of such minimum standards. HEC neither has the expertise for professional programs like medicine and dentistry, nor any mechanism is available to ensure uniformity of minimum standards at different universities and colleges across the country. One can only expect passing the bucket to others in case of non-observance of prescribed standards. This limited focus on the faculty of teaching institutions reveals how such decisions are far from being well-entrenched.

The performance of erstwhile PM&DC can be discussed as it too had miserably failed to perform its functions since it was politicized and both the major political parties of that time failed to come to some agreement and revive the PM&DC; however, reversal of its decisions by PMC in an unchecked and unbridled manner has led to a stir among healthcare fraternity. In one such development, PMC excluded M.Phil. Pathology from clinical qualification declaring it as an academic research-based program.^2^ The clarification by PMC further convoluted the matter.^3^

In yet another happening, PMC revealed that experience certificates shall be issued by the institution and not by PMC as was done by erstwhile PM&DC after verification and equivalence of experience against a standardized criterion.^4^ It further complicated the matter by advising to contact HEC or Medical Tribunal if an institution refuses to issue the experience certificate. Improving the existing standardized criteria and streamlining the promotion mechanism would have been a better preference than delegating it to institutions. Likewise, it was hardly a surprise when PMC took the stance that regulation of basic sciences will be dealt by HEC whereas those of clinical subjects will remain with PMC.

## The question remains:

What will be the short-term and long-term impact of such decisions? For few such decisions may be revolutionary; however, from the faculty’s standpoint, these have significant implications. In short, the PMC Act 2020, though has addressed certain challenges and provided legal cover, is still not fully comprehensive in its scope. It in no way manifests any opportunity to change discouraging trends and practices prevalent in the regulation of healthcare in Pakistan over seven decades. Nevertheless, PMC may rectify some of the chronic issues faced by medical education through need assessment of the on-ground situation, analysis by relevant experts, and consultation with all concerned stakeholders including the faculty.

## Authors’ Contribution:

**AE** wrote the first draft of the article which was subsequently revised by **AR**. Both authors approved the final submission.
